# Vitamin D deficiency in critically ill children: a systematic review and meta-analysis

**DOI:** 10.1186/s13054-017-1875-y

**Published:** 2017-11-23

**Authors:** James Dayre McNally, Nassr Nama, Katie O’Hearn, Margaret Sampson, Karin Amrein, Klevis Iliriani, Lauralyn McIntyre, Dean Fergusson, Kusum Menon

**Affiliations:** 1 Children’s Hospital of Eastern Ontario, 401 Smyth Road, Ottawa, ON K1H 8L1 Canada; 2 Division of Critical Care, Department of Pediatrics, Faculty of Medicine, University of Ottawa, Children’s Hospital of Eastern Ontario, 401 Smyth Road, Ottawa, ON K1H 8L1 Canada; 3 Faculty of Medicine, University of Ottawa, Ottawa, ON Canada; 4 Children’s Hospital of Eastern Ontario Research Institute, 501 Smyth Road, Ottawa, ON K1H 8L6 Canada; 5 Division of Endocrinology and Metabolism, Department of Internal Medicine, Medical University of Graz, Graz, Austria; 6 School of Medicine, Trinity College Dublin, Dublin, Ireland; 7 Division of Critical Care, Department of Medicine, Ottawa Hospital Research Institute (OHRI), University of Ottawa, Ottawa, ON Canada; 8 Department of Epidemiology and Community Medicine, Ottawa Hospital Research Institute (OHRI), University of Ottawa, Ottawa, Ontario Canada

**Keywords:** Vitamin D, Systematic review, Meta-analyses, Pediatrics, Mortality, Nutrition

## Abstract

**Background:**

Vitamin D deficiency (VDD) has been hypothesized not only to be common but also to represent a potentially modifiable risk factor for greater illness severity and clinical outcome during critical illness. The objective of this systematic review was to determine the frequency of VDD in pediatric critical illness and its association with clinical outcomes.

**Methods:**

MEDLINE, Embase, and CENTRAL were searched through December 12, 2016, with no date or language restrictions. The primary objective was to estimate the prevalence of VDD in the pediatric intensive care unit (PICU) and compare vitamin D status with healthy control populations. Secondary objectives were to evaluate whether VDD is associated with mortality, increased illness severity, PICU interventions, and patient clinical course. Random effects meta-analysis was used to calculate pooled VDD event rate, compare levels with those of control subjects, and evaluate for associations between VDD and clinical outcome.

**Results:**

Among 2700 citations, 17 studies meeting study eligibility were identified. The studies reported a total of 2783 critically ill children and had a median sample size of 120 (range 12–511). The majority of studies used a 25-hydroxyvitamin D [25(OH)D] level less than 50 nmol/L to define VDD, and the pooled VDD prevalence was 54.8 (95% CI 45.4–63.9). Average 25(OH)D levels were significantly lower in PICU patients than in healthy control subjects (pooled difference −17.3 nmol/L, 95% CI −14.0 to −20.6). In a meta-analysis calculation, we found that VDD was associated with increased mortality (OR 1.62, 95% CI 1.11–2.36), illness severity, and need for PICU interventions.

**Conclusions:**

Approximately 50% of critically ill children have VDD at the time of PICU admission, defined as a blood total 25(OH)D concentration under 50 nmol/L. VDD was further determined to be associated with greater illness severity, multiple organ dysfunction, and mortality in the PICU setting. Clinical trials are required to determine if optimization of vitamin D status improves patient outcome.

**Trial registration:**

PROSPERO, CRD42016026617. Registered on 11 January 2016.

**Electronic supplementary material:**

The online version of this article (doi:10.1186/s13054-017-1875-y) contains supplementary material, which is available to authorized users.

## Background

Severe vitamin D deficiency (VDD) is a well-established cause of disease, including hypocalcemia and skeletal abnormalities (e.g., rickets) [1–3]. Although severe deficiency causing classic bone manifestations is now rare, many adults and children endure a subclinical VDD state that may predispose them to neurologic, cardiovascular, respiratory, and immune pathology [4–6]. Because these organ systems are essential to the development of and recovery from critical illness, VDD has been hypothesized to be a risk factor for morbidity and mortality in the intensive care unit (ICU) [7].

Basic vitamin D physiology, specifically how the endocrine axis regulates calcium balance, is well described. Circulating 25-hydroxyvitamin D [25(OH)D], the inert precursor to the active hormone, is the accepted marker of body vitamin D status [8, 9]. Although thresholds and terminology vary, VDD is generally accepted as a 25(OH)D concentration below 50 nmol/L, with severe deficiency developing at 25–30 nmol/L [10–14]. These thresholds are based on both biochemical indicators of axis stress and values below which symptoms and disease predisposition rise. Briefly, when 25(OH)D falls into the 50 nmol/L range, maintenance of active hormone levels requires elevation of serum parathyroid hormone and increased renal enzyme activity [15, 16]. As 25(OH)D falls into the 30 nmol/L range, production of active hormone begins to fall, and healthy individuals can develop electrolyte disturbances and clinically evident disease related to inadequate blood and body calcium (rickets, seizures, myocardial disease) [16–18]. Although overt clinical disease is not evident in otherwise healthy individuals until 25(OH)D values drop below 30 nmol/L, population-based research has established improved bone health with 25(OH)D values over 50 nmol/L [10]. In addition to impaired calcium regulation within the gastrointestinal, renal, and skeletal systems, there are other mechanisms through which VDD could contribute to organ dysfunction in the ICU patient. For example, vitamin D is known to be essential for proper cardiovascular health, both indirectly through calcium and by controlling cell function directly via vitamin D receptors (VDRs) present on myocytes and endothelial cells. As a second example, there are functional VDRs present on all major immune cell types, and VDD has been implicated in proinflammatory states [19–21] and with impaired innate immunity [22–24]. As a consequence of these mechanisms and others including potential roles in skeletal myopathy and kidney disease [25–28], critical care physicians and researchers have hypothesized that VDD could lead to poorer outcome in the ICU setting.

Over the past 10 years, there have been dozens of observational studies evaluating vitamin D status in adult critical care settings, with recent meta-analyses calculating VDD to be associated with an almost twofold increased risk of death [29–31]. Because vitamin D status is rapidly modifiable, researchers have followed up these findings with multiple pilot clinical trials [32–38] and a single phase III randomized controlled trial (RCT) suggesting benefit derived from rapid normalization through enteral loading therapy [39]. Three meta-analyses of interventional vitamin D trials in critically ill adults have been published since 2016, further emphasizing the current relevance of this topic [40–42]. Unfortunately, owing to the small number of total evaluable patients and heterogeneity in study populations, these meta-analyses could only suggest potential benefit to supplementation, and they have set the stage for large phase III clinical trials in the adult ICU setting [43].

In recent years, multiple research groups have evaluated vitamin D status in critically ill children. However, owing to small sample sizes, the majority of these studies have lacked the power to sufficiently evaluate the relationship between vitamin D status, illness severity, and clinical course. Our objective here was to conduct a systematic review and meta-analysis to overcome these limitations. Our primary objective was to estimate the prevalence of VDD in the pediatric intensive care unit (PICU), and compare vitamin D status with that in healthy control populations. Secondarily, we sought to evaluate whether VDD is associated with mortality, increased illness severity, ICU interventions, and clinical course. This study will help inform the field regarding the need for further observational work and whether interventional trials should be entertained.

## Methods

The study protocol and objectives were established a priori (PROSPERO protocol registration number, CRD42016026617) and reported according to the Preferred Reporting Items for Systematic Reviews and Meta-Analyses guidelines [44] (Additional file 1).

### Eligibility criteria

Studies were included if the following eligibility criteria were met: (1) observational cohort or case-control study; (2) described a hospitalized pediatric population; (3) study participants were admitted to the PICU; (4) reported on vitamin D status, determined by total 25(OH)D; and (5) reported at least one of the following outcomes: mortality, mechanical ventilation, use of vasoactive agents, or PICU illness severity score. Mortality was the primary clinical outcome. A study was considered to be of pediatric subjects if it included patients younger than 18 years of age and did not include subjects over 21 years old. Studies in which researchers reported on populations admitted to both the PICU and the general ward were included as long as all other criteria were met. Studies were excluded if they (1) were case reports or case series (fewer than ten patients), (2) performed vitamin D measurement after death (i.e., study on sudden infant death), (3) included adults but did not report study findings separately for children, and (4) were focused on specific diseases or interventions (i.e., cardiac surgery, prematurity or very low birth weight, acute lower respiratory tract infection, hematopoietic stem cell transplant). Interventional studies were not considered, because a recent scoping review of pediatric clinical trials (with an online searchable database) did not identify any potentially relevant publications [45, 46].

### Data sources and study selection

The following databases were searched: MEDLINE, including in-process and other nonindexed citations (1946–December 12, 2016); PubMed to December 24, 2015; Embase (1974–December 21, 2015), and the Cochrane Central Register of Controlled Trials (CENTRAL) (November 2015 issue, searched December 21, 2015). MEDLINE, Embase, and CENTRAL were searched using the Ovid interface. The MEDLINE search strategy was developed by a librarian (MS) and peer-reviewed by a second librarian (Janet Joyce) using the Peer Review of Electronic Search Strategies standard [47]. The MEDLINE search was adapted for the other databases (Additional file 2). No date, language, or study design limits were applied. The search included a citation review of all eligible articles. Study eligibility was determined through two screening levels (Additional file 3). Title and abstract screening was performed by four authors (JDM, NN, KO, KI), followed by full-text review of potentially relevant citations by two independent authors (JDM, NN) using an online platform as previously described [45]. Disagreements between reviewers were resolved by consensus. Eligible studies were reviewed to identify duplicate reporting of study populations.

### Data extraction and risk of bias assessment

Data were extracted from eligible articles by one author and independently verified by a second, using a piloted and tested case report form on the REDCap platform (Research Electronic Data Capture) [48]. When necessary, data were extracted from figures using software (Digitizeit digitizer software; http://www.digitizeit.de/) [49]. Study methodological quality was evaluated using the Newcastle-Ottawa Scale (Additional file 4) as previously described [50, 51]. Countries were categorized as developed for subgroup analyses if they were classified as Very High Human Development according to the United Nations Human Development Report 2016, which is based on the Human Development Index [52]. Author groups were contacted for missing mortality data or to request the number of children with VDD using 50 nmol/L as the cutoff if another threshold was reported in the article.

### Data analysis and reporting

Random effects meta-analyses with inverse variance weighting were used to calculate pooled estimates and 95% CIs. Heterogeneity was assessed clinically and statistically using the *I* ^2^ statistic. For dichotomous outcomes, including the primary outcome, the event rates between groups were compared using OR. Continuous variables groups were compared through standard mean differences and their 95% CIs. If required, mean and SD values were determined from medians and IQRs [53]. Meta-analysis and metaregression were performed using Comprehensive Meta-Analysis version 3 software (Biostat, Englewood, NJ, USA).

## Results

### Literature search

**Fig. 1:**
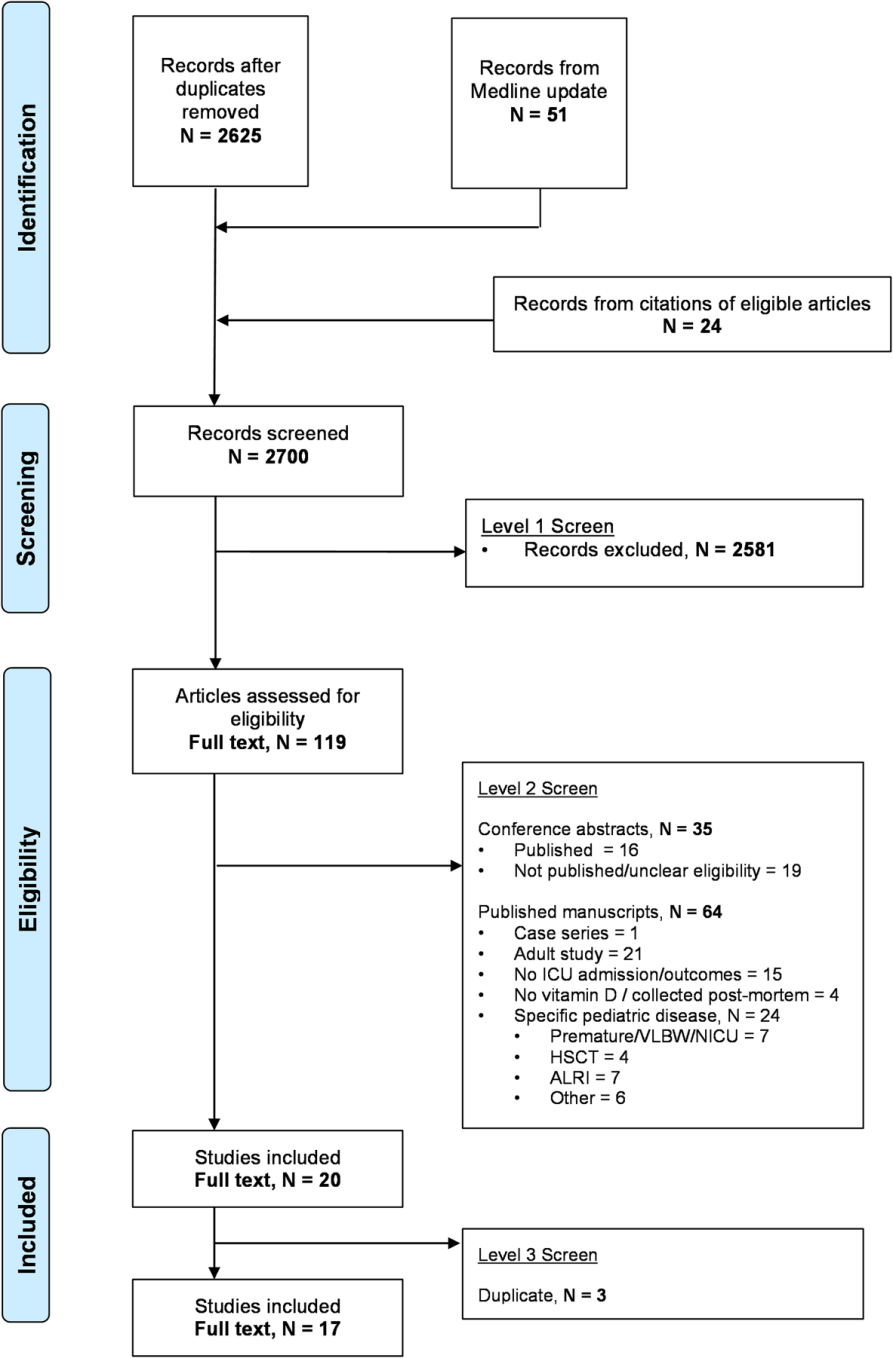
Preferred Reporting Items for Systematic Reviews and Meta-Analyses (PRISMA) flow diagram of study selection based on inclusion and exclusion criteria. The stages of a systematic selection scheme include identification, screening, eligibility, and final included studies. *ICU* Intensive care unit, *VLBW* Very low birth weight, *NICU* Neonatal intensive care unit, *HSCT* Hematopoietic stem cell transplant, *ALRI* Acute lower respiratory infection

There were 2700 unique records identified for screening (Fig. 1). Following abstract screening, 119 articles were retained, with 20 articles meeting full eligibility criteria [54–73]. Three articles were identified as secondary analyses of previous publications [59, 61, 68].

### Description of included studies

Study characteristics, geographical origin, major inclusion and exclusion criteria, and study size are shown in Table 1. With the exception of the 1990 report by Gauthier et al. [54], all studies were published after September 2012. Studies originated in eight different countries, with 41% (*n* = 7) originating in developing countries (India = 6, Thailand = 1). Only two studies recruited children from more than one PICU, including six sites in a Canadian study and two in an Irish study [62, 63]. Altogether, the studies reported on 2783 children, with a median size of 120 (range 12–511).

**Table 1:** Characteristics of study setting, population, and vitamin D levels

First author, year [reference]	Country	Age (major inclusions)	Major exclusions^a^	Sample size	NOS	Control population
Gauthier, 1990 [54]	United States	Under 18 years	Renal	45	4	Yes
Madden, 2012 [60]	United States	Under 21 years	Postoperative (cardiac)	511	9	No
McNally, 2012 [62]	Canada	Under 17 years	None	326	8	No
Rippel, 2012 [69]	Australia	Age not specified	Liver, renal, bone, 22q11	316	8	No
Ayulo, 2014 [55]	United States	1–21 years	None	216	6	No
Dayal, 2014 [66]	India	0.25–12 years	Renal, liver, malabsorption	92	7	No
Hebbar, 2014 [57]	United States	0–18 years	Renal, malabsorption, postoperative (elective)	61	6	Yes
Rey, 2014 [65]	Spain	Under 16 years	None	156	8	Yes
Korwutthikulrangsri, 2015 [58]	Thailand	0–18 years	Liver	32	7	Yes
Onwuneme, 2015 [63]	Ireland	Under 12 years (sepsis)	Postoperative (cardiac)	120	8	Yes
Prasad, 2015 [64]	India	2 months to 12 years (medical)	None	80	8	No
Ebenezer, 2016 [56]	India	Age not specified	None	52	7	No
Ponnarmeni, 2016 [67]	India	1–12 years (sepsis)	Preexisting disease, vitamin D	124	7	Yes
Bustos, 2016 [71]	Chile	0–15 years	Liver, kidney disease	90	9	No
García-Soler, 2017 [70]	Spain	6 months to 15 years	Renal, parathyroid, malabsorption, vitamin D	340	8	No
Sankar, 2016 [73]	India	1 month to 17 years	Renal, rickets, vitamin D	101	8	No
Shah, 2016 [72]	India	1 month to 15 years	Rickets, parathyroid, renal	154	6	No

*NOS* Newcastle-Ottawa Scale

^a^Major exclusions considered were admission categories (medical, surgical); vitamin D supplementation; renal, parathyroid, or liver disease; malabsorption syndrome; rickets; or genetic conditions associated with impaired vitamin D axis (e.g., 22q11)

All studies evaluated a general PICU population, with two focused on patients with sepsis [63, 67], one on medical admissions [64], and another on a mixed ward and ICU cohort [66]. Common major exclusion criteria were renal disease (*n* = 8), liver disease (*n* = 4), malabsorption syndromes (*n* = 3), and cardiac surgery (*n* = 2). Risk of bias assessment showed a median study score of 8 (range 4–9) for the Newcastle-Ottawa Scale (Table 1). The most common reason leading to a reduction in the Newcastle Ottawa Scale was lack of comparability, with only seven studies (41%) receiving full marks [60, 62, 63, 69–71, 73].

### Study objectives

A single well-defined primary objective was provided for five studies and in all instances focused on a measure of vitamin D status. Of the remaining studies, 11 had a combined objective to evaluate vitamin D status and relationship with risk factors (*n* = 2), illness severity (*n* = 2), adrenal function (*n* = 1), positive sepsis culture rate (*n* = 1), or clinical outcome (*n* = 6). The remaining study had a general objective of understanding calcium metabolism [54].

### Vitamin D status

**Fig. 2:**
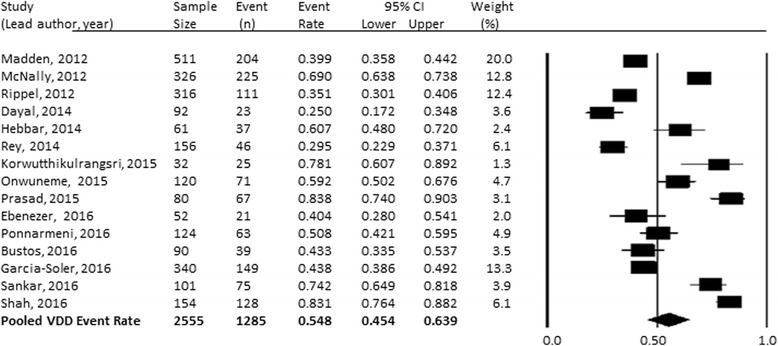
Pooled vitamin D deficiency event rate using 50 nmol/L as the threshold to define vitamin D deficiency

Vitamin D status and/or VDD prevalence for both the critically ill cohort and control populations are shown in Table 2. All of the studies determined vitamin D status at admission (or first day). Average vitamin D level (mean or median) was provided or could be calculated for 16 studies (range 14.5–72 nmol/L). The most commonly used threshold to define VDD was 50 nmol/L (15 studies), with the exception of Ayulo et al. (37.5 nmol/L) [55] and Gauthier et al. (22.5 nmol/L) [54]. Among the 15 studies using the 50 nmol/L threshold, the median VDD prevalence rate was 51% (range 25–84%), and the pooled VDD event rate was 54.8 (95% CI 45.4–63.9) (*see* Fig. 2). Studies done in developing countries had a higher pooled VDD rate (64%, 95% CI 51–75%) than those done in developed countries (47%, 95% CI 36–59%), although the difference was not statistically significant (*p* = 0.52).

**Table 2:** Vitamin D status at pediatric intensive care unit admission (or first day in pediatric intensive care unit) for critically ill children and healthy control subjects

First author, year [reference]	Threshold VDD (nmol/L)	PICU cohort			Control cohort		
		%VDD	Average 25(OH)D (nmol/L)^a^	Mortality % (*n*/*N* ^b^)	(*N* ^c^)	%VDD	Average 25(OH)D (nmol/L)^a^
Gauthier, 1990 [54]	22.5	NR	66.8^d^ (±20)	11 (5/45)	12	NR	82.5^d^ (±25)
Madden, 2012 [60]	50	40	56.3 (41–78.3)	3 (13/511)			
McNally, 2012 [62]	50	69	43.2^d^ (±19.4)	2 (5/326)			
Rippel, 2012 [69]	50	34	56.6 (44–70)	3 (10/316)			
Ayulo, 2014 [55]	37.5^e^	28	NR	3 (6/216)			
Dayal, 2014 [66]	50	25	71.9^d^ (±27.3)	7 (6/92)			
Hebbar, 2014 [57]	50	61	59^d^ (±43)	NR	42	23.9	99^d^ (±55)
Rey, 2014 [65]	50	30	65 (48–89.5)	3 (4/156)^f^	289	16%	76.3 (58–96.5)
Korwutthikulrangsri, 2015 [58]	50	78	41.5 (33.3–48.8)	19 (6/32)	36	19.5	61^d^ (±12)
Onwuneme, 2015 [63]	50	59	47^d^ (±29)	2 (2/120)^f^	30	NR	66^d^ (±26)
Prasad, 2015 [64]	50	84	30.3 (22.5–45)	19 (15/80)			
Ebenezer, 2016 [56]	50	40	62.8 (40.5–85.5)	19 (10/52)			
Ponnarmeni, 2016 [67]	50	51	49.3^d^ (±30)	15 (19/124)	338	40.2%	68.7^d^ (±40)
Bustos, 2016 [71]	50	43	57.0^d^ (±24)	4 (4/90)			
García-Soler, 2017 [70]	50	44	57.0^d^ (±26)	3 (10/340)			
Sankar, 2016 [73]	50	74	14.5 (10–20)	31 (31/101)			
Shah, 2016 [72]	50	83	29.3 (7–40)	44 (68/154)			

*Abbreviations*: *25(OH)D* 25-Hydroxyvitamin D, *NR* Not reported, *PICU* Pediatric intensive care unit, *VDD* Vitamin D deficiency

^a^Average reported as median with IQR unless otherwise specified as mean

^b^Total number of PICU patients enrolled for which a measure of vitamin D status was available

^c^Total number of control patients enrolled for which a measure of vitamin D status was available

^d^With SD (±SD)

^e^Did not respond to a request for vitamin D status and outcome data categorized using the 50 nmol/L threshold

^f^Data provided by authors outside of publication

### Comparison with control subjects

As shown in Table 2, researchers in six studies investigated whether critically ill children had lower vitamin D than a control population [54, 57, 58, 63, 65, 67]. A significantly lower 25(OH)D level was calculated for PICU patients than for healthy control subjects (pooled mean difference −17.3 nmol/L, 95% CI −14.0 to −20.6, *p* = 0.001) (Additional file 5).

### Baseline demographics and risk factors

With the exception of Gauthier et al. [54], researchers in all studies investigated the relationship between vitamin D status and at least one risk factor (Additional file 6). Factors identified as significantly associated with lower vitamin D levels in more than one study included weight (*n* = 4) [55, 62, 65, 70] (i.e., body mass index [BMI]), lower nutritional status (*n* = 6) [55, 62, 65–67, 70] (i.e., failure to thrive), older age (*n* = 6) [55, 56, 60, 65, 70, 72], admission type (*n* = 4) [62, 65, 69, 70] (i.e., nontrauma, cardiac, metabolic/renal), seasons with decreased ultraviolet light exposure (*n* = 5) [57, 60, 70–72], non-Caucasian race (*n* = 4) [55, 57, 60, 70], and absence of supplementation (*n* = 2) [60, 63].

### Vitamin D status and mortality

**Fig. 3:**
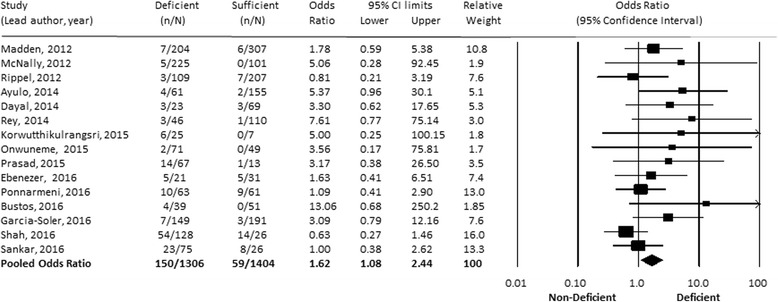
Association of vitamin D deficiency and mortality. The pooled OR was also statistically significant when analyzed using the Mantel-Haenszel or Peto OR

**Fig. 4:**
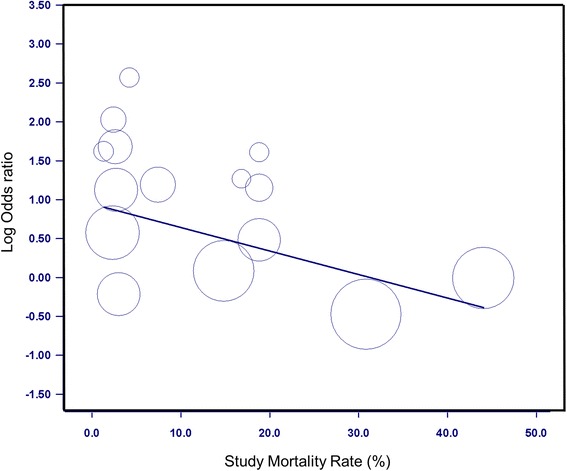
Interaction between overall study mortality rate and impact of vitamin D deficiency on mortality. Metaregression analysis confirming the interaction between baseline mortality rate and impact of vitamin D deficiency on mortality. Significantly higher odds of death were evident in patients with vitamin D deficiency originating from studies with lower mortality (*p* = 0.02). Size of the data markers indicates the weight of the study, with larger circles indicating smaller studies

Mortality data were available in 13 publications, with 2 groups responding to a request for results [63, 65]. The final mortality data included 2710 children and 210 deaths, with study mortality ranging from 1.5% to 44%. The pooled mortality event rate was calculated at 7.9% (95% CI 1–45%), with statistically higher rates in developing countries (pooled event rates, 21% vs 3%; *p* < 0.001). Critically ill children with VDD had significantly higher mortality than critically ill children without VDD (pooled OR 1.62, 95% CI 1.07–2.44, *p* = 0.02) (Fig. 3). Removing the one study in which researchers reported on a mixed cohort of ward and PICU patients [66] did not change this result. Evaluating the relationship by country of origin, we determined that VDD was statistically associated with mortality in developed countries (OR 2.56, 95% CI 1.38–4.6, *p* = 0.003) but not in developing countries (OR 1.12, 95% CI 0.71–1.78), with the difference between groups achieving statistical significance (*p* = 0.03) (Additional file 7). Metaregression further identified that the relationship between VDD and mortality was significantly influenced by the study mortality rate (intercept 0.955, 95% CI 0.38–1.53; baseline mortality −0.030, 95% CI −0.055 to −0.0054; *p* = 0.017) (Fig. 4). When we repeated the mortality analysis, restricting it to those studies with Newcastle-Ottawa Scale scores of 8 or above (*n* = 9 of 15), the random effects OR was similar, with a slight increase in OR to 1.82 (95% CI 1.08–3.06, *p* = 0.02).

### Vitamin D status and illness severity

Fifteen studies investigated the relationship between vitamin D status and an illness severity or organ dysfunction score, including Pediatric Risk of Mortality (PRISM III) (*n* = 9), Pediatric Index of Mortality (PIM2) (*n* = 5), pediatric logistic organ dysfunction score (*n* = 4), and Sequential Organ Failure Assessment (*n* = 2) (Table 3). Relationship with illness severity was most often investigated through comparison of scores between VDD and nondeficient groups (*n* = 13). Analyses of the five studies reporting an illness severity score by group with mean and SD values demonstrated higher scores in the VDD group (pooled standardized mean difference 0.43, 95% CI 0.29–0.57, *p* = 0.001). The results remained significant when we incorporated calculated results from eight additional studies reporting median and IQR values (pooled standardized mean difference 0.24, 95% CI 0.04–0.44, *p* = 0.02). In the two studies in which researchers evaluated the relationship using correlation or regression approaches, a statistically significant inverse relationship between 25(OH)D levels and PIM2 [56] or PRISM III [60] was reported.

**Table 3:** Illness severity, organ dysfunction, and clinical outcomes

First author, year [reference]	Mortality	MV	Vasoactive	Illness severity/organ dysfunction	Ca^2+^	Hospital/PICU LOS	Other outcomes
Gauthier, 1990 [54]	No	No	No	No	Yes	No	None
Madden, 2012 [60]	Yes	Yes	Yes/SOFA	PRISM III	No	No	Fluid, severe septic shock, positive culture
McNally, 2012 [62]	Yes	Yes	Yes	PRISM III	Yes	PICU	Fluid bolus
Rippel, 2012 [69]	Yes	Yes	Yes	PIM2	Yes	PICU/Hospital	ECMO, hypotension, arrhythmia, arrest
Ayulo, 2014 [55]	Yes	No	No	PELOD^a^	Yes	No	Fluid
Dayal, 2014 [66]	Yes	Yes	Yes	No	No	Hospital	Nosocomial sepsis
Hebbar, 2014 [57]	No	No	Yes	PRISM III, PELOD, SOFA	No	No	Sepsis, shock, antimicrobial peptide
Rey, 2014 [65]	No^a^	Yes	Yes	PRISM III/PIM2	No	PICU	Platelets, CRP
Korwutthikulrangsri, 2015 [58]	Yes	Yes	Yes	PRISM III	Yes	PICU	Septicemia, shock, adrenal insufficiency, CRP
Onwuneme, 2015 [63]	No^a^	Yes	Yes	PIM2	No	PICU	Shock, fluid, culture-positive sepsis, CRP, platelets, RRT
Prasad, 2015 [64]	Yes^b^	Yes	Yes^b^	PRISM III	Yes	PICU/Hospital^b^	Coagulopathy, culture-positive sepsis
Ebenezer, 2016 [56]	Yes	Yes	Yes/SOFA	PIM2	Yes	PICU	Positive culture, CRP
Ponnarmeni, 2016 [67]	Yes	Yes	Yes	PRISM III/SOFA/MODS	Yes	PICU	Septic shock, positive culture
Bustos, 2016 [71]	Yes	Yes	Yes	PRISM III/PELOD	Yes	PICU/Hospital	Septic shock, fluid, RRT, CRP, PCT, platelets
García-Soler, 2017 [70]	Yes	Yes	Yes	PRISM III	Yes	PICU	Platelets, CRP, PCT, full course of antibiotics, morbidity^c^
Sankar, 2016 [73]	Yes	Yes	Yes	PIM2/PELOD	Yes	PICU	Fluid
Shah, 2016 [72]	Yes	Yes	No	PIM2	Yes	PICU	Severe sepsis, liver failure, ARDS, RRT
Total	13	14	14	15	12	12/3	Positive culture (*n* = 5), CRP (*n* = 6), sepsis/shock (*n* = 8)

*Abbreviations*: *ARDS* Acute respiratory distress syndrome, *CRP* C-reactive protein, *ECMO* Extracorporeal membrane oxygenation, *LOS* Length of stay, *MV* Mechanical ventilation, *PICU* Pediatric intensive care unit, *PELOD* Pediatric logistic organ dysfunction score, *PRISM III* Pediatric Risk of Mortality, *SOFA* Sequential Organ Failure Assessment, *RRT* Renal replacement therapy, *PCT* Procalcitonin

^a^Responded to request for mortality data

^b^Comment only; data not shown

^c^Had a composite morbidity outcome that included mortality, use of vasoactive agents for longer than 24 h, more than 7 days of antibiotics, need for parenteral nutrition, continuous renal replacement therapy

### Vitamin D status and cardiovascular support

Administration of a vasoactive agent (i.e., catecholamines and milrinone) for cardiovascular support was one of the three most commonly reported ICU interventions (*n* = 14) (Table 3). It was possible to pool findings from ten studies that provided data as a dichotomous outcome (required/not required), and significantly increased rates of vasoactive agent use (pooled OR 1.97, 95% CI 1.49–2.61, *p* < 0.001) were observed in the VDD group (Table 4, Additional file 8). Removing any of the 11 individual studies from the analysis did not significantly change the OR or impact statistical significance (*p* < 0.001 for all).

**Table 4:** Relationship between vitamin D deficiency status and markers of illness severity and intensive care unit outcome

ICU outcome or intervention	Pooled OR (95% CI)			
	All studies	Developed countries	Developing countries	*p* Value^a^
Mortality	1.62 (1.08–2.44)	2,52 (1.37–4.60)	1.12 (0.71–1.78)	0.04
Mechanical ventilation	1.83 (1.28–2.63)	1.92 (1.23–3.01)	1.69 (0.85–3.37)	0.74
Vasopressor use	1.97 (1.49–2.61)	2.22 (1.54–3.21)	1.52 (0.93–2.50)	0.29
Infection (bacterial/nosocomial)	2.21 (1.50–3.25)	2.49 (1.41–4.38)	1.77 (0.94–3.33)	0.55

Pooled OR from random effects meta-analysis evaluating the relationship between vitamin D status and established markers of illness severity, intervention, and outcome in the pediatric intensive care unit setting

^a^Metaregression was used to evaluate whether the pooled ORs were statistically different between developed and developing countries

### Vitamin D status and mechanical ventilation

Fourteen studies provided data on mechanical ventilation (Table 3), and mechanical ventilation was most frequently reported as a requirement for any form of invasive ventilation, excluding noninvasive ventilation. Significantly increased rates of mechanical ventilation (pooled OR 1.83, 95% CI 1.28–2.63, *p* < 0.001) were observed in the VDD group (Table 4, Additional file 9). Repeating the analysis with any of the individual studies removed did not significantly change the OR or statistical significance.

### Vitamin D status and PICU length of stay

PICU length of stay was reported in 11 studies (Table 3). Although the relationship between VDD and PICU or hospital length of stay was evaluated in 11 studies, only 3 of them [62, 66, 73] provided data in a format that allowed calculation of a pooled difference in means (1.99 days, 95% CI 0.88–3.10, *p* = 0.001). The pooled difference in means remained statistically significant if any individual study was removed from the analysis (*p* < 0.02).

### Vitamin D status and infection

The relationship between vitamin D status and infection was reported in five studies. Data analyses identified children with VDD to have a statistically increased likelihood of confirmed bacterial or nosocomial infection (pooled OR 2.21, 95% CI 1.50–3.24, *p* < 0.001) (provided in Additional file 10). Given clinical heterogeneity in infection definition (Additional file 10), we performed one-study-removed sensitivity analysis, and all pooled ORs remained highly statistically significant (*p* < 0.001). When the analysis was further restricted to the four studies using a similar definition (culture-positive), the findings remained statistically significant (pooled OR 2.02, 95% CI 1.08–3.78, *p* = 0.03).

## Discussion

To our knowledge, this is the first systematic review evaluating vitamin D status in critically ill children. We identified 17 studies from 8 countries and 5 continents. The worldwide prevalence of VDD was calculated as 54% at the time of PICU admission, with deficiency associated with greater illness severity, use of ICU interventions, and mortality.

Evaluation of vitamin D status was the primary objective in most studies. VDD was most commonly defined as 25(OH)D under 50 nmol/L. By combining data from 2555 children, we were able to generate a robust estimate of the VDD event rate (54%) in critically ill children. Although a small difference was observed between developed (47%) and developing (64%) countries, the risk of VDD remained high regardless of geography. Importantly, multiple studies also provided vitamin D levels for a control population, with meta-analyses concluding that critically ill children have lower 25(OH)D, by an average of 17 nmol/L. In addition, indirect comparison with large national population-based studies also suggests vitamin D levels to be lower. For example, the Canadian Health Measures Survey estimated the mean concentration of 25(OH)D to be 75 nmol/L among children aged 6–11 years, and a separate study of preschoolers estimated that 88% had 25(OH)D levels above 50 nmol/L [74, 75]. Similarly, estimates from national surveys done in the United States and European countries also suggest average 25(OH)D concentration to be near 70 nmol/L, with only 20% having levels under 50 nmol/L [76, 77]. In contrast, adult ICU studies show comparable vitamin D levels, with reported mean 25(OH)D levels ranging from 13 to 62 nmol/L and with 80% of studies since 2009 identifying average group levels below 50 nmol/L [78]. Despite the established rate of VDD in the ICU and associations with poorer outcome, there is still insufficient evidence on which to base supplementation guidelines specific to the critically ill population [79, 80]. Accordingly, the prevalence of VDD in the ICU remains high.

This review also sought to investigate the relationship of VDD with illness severity, ICU interventions, and clinical outcomes. We focused on mortality as the primary clinical outcome because it is a commonly reported objective measure amenable to meta-analysis that is accepted as meaningful [81, 82]. Mortality data were available for 15 studies and 2710 patients, representing 97% of the total cohort. Individual studies lacked adequate power to evaluate the relationship between VDD and mortality, owing to small sample size. For example, although four studies reported all deaths in the VDD group [58, 62, 63, 71], the findings achieved statistical significance only in the Chilean study [71]. The association with mortality was stronger and achieved greater statistical significance (OR 2.6, *p* = 0.003) when developing countries were removed. Our findings are consistent with the results from systematic reviews by de Haan et al. [29] (RR 1.7, 95% CI 1.49–2.16) and Zhang et al. [30] (OR 1.76, 95% CI 1.38–2.24) evaluating the same question in the adult ICU setting. There are multiple biologically plausible mechanisms through which VDD could influence the development and recovery from critical illness, including calcium homeostasis and the stress response of nonclassical organs, including the immune, cardiac, and respiratory systems [83–85]. Our study findings further support the pleiotropic nature of the hormone, with significant associations between VDD and mechanical ventilation, vasopressor use, and confirmed bacterial or nosocomial infection.

A common question in this area of research relates to the mechanisms leading to low blood concentrations of vitamin D in critical illness. In the ambulatory setting, the risk factors are well defined and include impaired skin synthesis, restricted dietary intake, and genetics [14, 85–87]. Some PICU studies incorporated these variables and were able to confirm that factors such as such as season of presentation [57, 60], absent vitamin D supplementation [60, 63], non-Caucasian race [55, 57, 60], and obesity [66, 67] were associated with greater risk of VDD among critically ill children. Unfortunately, the available studies were not designed to fully explain why critically ill children as a whole have considerably lower vitamin D levels. It has been suggested that altered metabolism and acute care interventions may rapidly lower blood vitamin D concentration. This has been confirmed in some targeted studies evaluating levels before and after surgeries, extracorporeal interventions, and conditions associated with severe inflammation [88–90]. Consequently, many researchers have cautioned against overinterpreting observational study findings, suggesting that confounding factors may be driving the relationship between vitamin D status and outcome [91].

Regardless of how critically ill children arrive at their vitamin D-deficient state, VDD may contribute to secondary pathophysiology. Furthermore, restoring blood concentrations has the potential to facilitate clinical recovery in the PICU and presents a target question for interventional trials. The present systematic review helps to pave the way for future RCTs of vitamin D supplementation in critically ill children by guiding outcome measure selection. Our results suggest that mortality may not be the best choice for a primary outcome, owing to the low event rate (less than 5%) in developed countries and lack of significant association in developing countries. Because our systematic review findings also suggested pleiotropic actions of vitamin D, it would be reasonable to consider a composite outcome of mortality and faster resolution of organ dysfunction (e.g., PICU stay) or postillness health-related quality of life.

Although based on an exhaustive literature search and comprehensive synthesis effort, this review has limitations. First, the findings are based on data derived from observational data, so one should be careful not to draw conclusions on causation, because the relationship may be driven by confounding factors. Second, although total serum 25(OH)D is well accepted as the best marker of vitamin D status in stable outpatient populations, some uncertainty remains regarding whether an alternative assay [e.g., free 25(OH)D or active hormone] might better define VDD in the ICU [59, 68]. Third, although 50 nmol/L is well accepted as a legitimate threshold for defining VDD, some evidence shows that the benefit of supplementation, or type of benefit, may be limited to lower thresholds (e.g., below 30 nmol/L) [39, 61]. Because few studies reported 25(OH)D levels using alternate thresholds for severe deficiency, it was not possible to evaluate whether the relationship of VDD to PICU outcomes was more significant in this subgroup. Finally, only a minority of studies controlled for relevant patient characteristics in their investigation of the relationship between VDD and clinical outcome. To contribute significantly, any further observational studies should be adequately designed and powered to consider covariates in their analyses.

## Conclusions

In this systematic review, we identified VDD to be highly prevalent in the PICU and to be associated with illness severity and clinical outcome. Benefits of optimization of vitamin D status need to be addressed in RCTs. This review not only provides a rationale for an RCT but also contributes important information for the selection of an outcome measure. Recognizing that we were unable to consider alternative assays (i.e. free hormone), metabolites (i.e. active hormone), and thresholds to define VDD in this systematic review, we feel it will be important that future RCTs incorporate these variables into their analyses.

## Additional files

Additional file 1: PRISMA checklist. (PDF 333 kb)

Additional file 2: Electronic search strategy used for this systematic review. (PDF 452 kb)

Additional file 3: Table summarizing the screening criteria used for level 1 and level 2 screening. (PDF 165 kb)

Additional file 4: Newcastle-Ottawa Scale adapted for this systematic review. (PDF 352 kb)

Additional file 5: Comparison of vitamin D status at PICU admission (or first day in PICU) of critically ill children and healthy control subjects. Table summarizing vitamin D status at PICU admission (or first day in PICU) of critically ill children and healthy control subjects, and the pooled mean difference in 25(OH)D levels between the PICU and control cohorts. (PDF 78 kb)

Additional file 6: Table summarizing the demographics and risk factors for vitamin D deficiency evaluated by each study included in this systematic review. (PDF 77 kb)

Additional file 7: Association of vitamin D deficiency and mortality by country type. Figure showing association of vitamin D deficiency and mortality in developed and developing countries. (TIF 218 kb)

Additional file 8: Vitamin D deficiency and clinical outcomes in the PICU. Figure showing association of vitamin D deficiency with vasopressor use. (TIF 74 kb)

Additional file 9: Vitamin D deficiency and clinical outcomes in the PICU. Figure showing association of vitamin D deficiency with mechanical ventilation. (TIF 74 kb)

Additional file 10: Vitamin D deficiency and clinical outcomes in the PICU. Figure showing association of vitamin D deficiency with confirmed bacterial or nosocomial infection. (TIF 175 kb)

## Abbreviations

25(OH)D: 25-Hydroxyvitamin D
ALRI: Acute lower respiratory infection
BMI: Body mass index
CRP: C-reactive protein
ECMO: Extracorporeal membrane oxygenation
HSCT: Hematopoietic stem cell transplant
ICU: Intensive care unit
LOS: Length of stay
MV: Mechanical ventilation
NICU: Neonatal intensive care unit
NOS: Newcastle-Ottawa Scale
NR: Not reported
PCT: Procalcitonin
PELOD: Pediatric logistic organ dysfunction score
PICU: Pediatric intensive care unit
PIM2: Pediatric Index of Mortality
PRISM: Pediatric Risk of Mortality
PRISMA: Preferred Reporting Items for Systematic Reviews and Meta-Analyses
RCT: Randomized controlled trial
REDCap: Research Electronic Data Capture
RRT: Renal replacement therapy
SOFA: Sequential Organ Failure Assessment
VDD: Vitamin D deficiency
VDR: Vitamin D receptor
VLBW: Very low birth weight

## References

[1] Mehrotra P, Marwaha RK, Aneja S, Seth A, Singla BM, Ashraf G, Sharma B, Sastry A, Tandon N (2010). Hypovitaminosis D and hypocalcemic seizures in infancy. Indian Pediatr.

[2] Basatemur E, Sutcliffe A (2015). Incidence of hypocalcemic seizures due to vitamin D deficiency in children in the United Kingdom and Ireland. J Clin Endocrinol Metab.

[3] Ward LM, Gaboury I, Ladhani M, Zlotkin S (2007). Vitamin D-deficiency rickets among children in Canada. CMAJ.

[4] Zittermann A, Gummert JF (2010). Nonclassical vitamin D action. Nutrients.

[5] Kim SY (2013). The pleiomorphic actions of vitamin D and its importance for children. Ann Pediatr Endocrinol Metab.

[6] Ginde AA, Mansbach JM, Camargo CA (2009). Vitamin D, respiratory infections, and asthma. Curr Allergy Asthma Rep.

[7] Lee P, Nair P, Eisman JA, Center JR (2009). Vitamin D deficiency in the intensive care unit: an invisible accomplice to morbidity and mortality?. Intensive Care Med.

[8] Seamans KM, Cashman KD (2009). Existing and potentially novel functional markers of vitamin D status: a systematic review. Am J Clin Nutr.

[9] Institute of Medicine (1997). Dietary reference intakes for calcium, phosphorus, magnesium, vitamin D, and fluoride.

[10] Ross AC, Manson JE, Abrams SA, Aloia JF, Brannon PM, Clinton SK, Durazo-Arvizu RA, Gallagher JC, Gallo RL, Jones G (2011). The 2011 report on dietary reference intakes for calcium and vitamin D from the Institute of Medicine: what clinicians need to know. J Clin Endocrinol Metab.

[11] Wagner CL, Greer FR (2008). Prevention of rickets and vitamin D deficiency in infants, children, and adolescents. Pediatrics.

[12] Canadian Paediatric Society (2007). Vitamin D supplementation: recommendations for Canadian mothers and infants. Paediatr Child Health.

[13] Thacher TD, Clarke BL (2011). Vitamin D insufficiency. Mayo Clin Proc.

[14] Holick MF (2007). Vitamin D, deficiency. N Engl J Med.

[15] Willett AM (2007). Vitamin D, status and its relationship with parathyroid hormone and bone mineral status in older adolescents. Proc Nutr Soc.

[16] Christensen MH, Lien EA, Hustad S, Almas B (2010). Seasonal and age-related differences in serum 25-hydroxyvitamin D, 1,25-dihydroxyvitamin D and parathyroid hormone in patients from Western Norway. Scand J Clin Lab Invest.

[17] Docio S, Riancho JA, Pérez A, Olmos JM, Amado JA, González-Macías J (1998). Seasonal deficiency of vitamin D in children: a potential target for osteoporosis-preventing strategies?. J Bone Miner Res.

[18] Thacher TD, Fischer PR, Isichei CO, Pettifor JM (2006). Early response to vitamin D_2_ in children with calcium deficiency rickets. J Pediatr.

[19] Baeke F, Gysemans C, Korf H, Mathieu C (2010). Vitamin D insufficiency: implications for the immune system. Pediatr Nephrol.

[20] Rigby WF, Denome S, Fanger MW (1987). Regulation of lymphokine production and human T lymphocyte activation by 1,25-dihydroxyvitamin D_3_: Specific inhibition at the level of messenger RNA. J Clin Invest.

[21] Bhalla AK, Amento EP, Serog B, Glimcher LH (1984). 1,25-Dihydroxyvitamin D_3_ inhibits antigen-induced T cell activation. J Immunol.

[22] Hata TR, Kotol P, Jackson M, Nguyen M, Paik A, Udall D, Kanada K, Yamasaki K, Alexandrescu D, Gallo RL (2008). Administration of oral vitamin D induces cathelicidin production in atopic individuals. J Allergy Clin Immunol.

[23] Gombart AF, Borregaard N, Koeffler HP (2005). Human cathelicidin antimicrobial peptide (CAMP) gene is a direct target of the vitamin D receptor and is strongly up-regulated in myeloid cells by 1,25-dihydroxyvitamin D_3_. FASEB J.

[24] Jeng L, Yamshchikov AV, Judd SE, Blumberg HM, Martin GS, Ziegler TR, Tangpricha V (2009). Alterations in vitamin D status and anti-microbial peptide levels in patients in the intensive care unit with sepsis. J Transl Med.

[25] Al-Said YA, Al-Rached HS, Al-Qahtani HA, Jan MM (2009). Severe proximal myopathy with remarkable recovery after vitamin D treatment. Can J Neurol Sci.

[26] Ward KA, Das G, Berry JL, Roberts SA, Rawer R, Adams JE, Mughal Z (2009). Vitamin D status and muscle function in post-menarchal adolescent girls. J Clin Endocrinol Metab.

[27] van der Heyden JJ, Verrips A, ter Laak HJ, Otten B, Fiselier T (2004). Hypovitaminosis D-related myopathy in immigrant teenagers. Neuropediatrics.

[28] Torun E, Genc H, Gonullu E, Akovali B, Ozgen IT (2013). The clinical and biochemical presentation of vitamin D deficiency and insufficiency in children and adolescents. J Pediatr Endocrinol Metab.

[29] de Haan K, Groeneveld AB, de Geus HR, Egal M, Struijs A (2014). Vitamin D deficiency as a risk factor for infection, sepsis and mortality in the critically ill: systematic review and meta-analysis. Crit Care.

[30] Zhang YP, Wan YD, Sun TW, Kan QC, Wang LX (2014). Association between vitamin D deficiency and mortality in critically ill adult patients: a meta-analysis of cohort studies. Crit Care.

[31] Zajic P, Amrein K (2014). Vitamin D deficiency in the ICU: a systematic review. Minerva Endocrinol.

[32] Amrein K, Sourij H, Wagner G, Holl A, Pieber TR, Smolle KH, Stojakovic T, Schnedl C, Dobnig H (2011). Short-term effects of high-dose oral vitamin D_3_ in critically ill vitamin D deficient patients: a randomized, double-blind, placebo-controlled pilot study. Crit Care.

[33] Mata-Granados JM, Vargas-Vasserot J, Ferreiro-Vera C, de Castro MDL, Pavón RG, Quesada Gómez JM (2010). Evaluation of vitamin D endocrine system (VDES) status and response to treatment of patients in intensive care units (ICUs) using an on-line SPE-LC-MS/MS method. J Steroid Biochem Mol Biol.

[34] Nair P, Venkatesh B, Lee P, Kerr S, Hoechter DJ, Dimeski G, Grice J, Myburgh J, Center JR (2015). A randomized study of a single dose of intramuscular cholecalciferol in critically ill adults. Crit Care Med.

[35] Dickerson RN, Berry SC, Ziebarth JD, Swanson JM, Maish GO, Minard G, Brown RO (2015). Dose-response effect of ergocalciferol therapy on serum 25-hydroxyvitamin D concentration during critical illness. Nutrition.

[36] Han JE, Jones JL, Tangpricha V, Brown MA, Brown LA, Hao L, Hebbar G, Lee MJ, Liu S, Ziegler TR, Martin GS (2016). High Dose Vitamin D administration in ventilated intensive care unit patients: a pilot double blind randomized controlled trial. J Clin Transl Endocrinol.

[37] Leaf DE, Raed A, Donnino MW, Ginde AA, Waikar SS (2014). Randomized controlled trial of calcitriol in severe sepsis. Am J Respir Crit Care Med.

[38] Aminmansour B, Nikbakht H, Ghorbani A, Rezvani M, Rahmani P, Torkashvand M, Nourian M, Moradi M (2012). Comparison of the administration of progesterone versus progesterone and vitamin D in improvement of outcomes in patients with traumatic brain injury: a randomized clinical trial with placebo group. Adv Biomed Res.

[39] Amrein K, Schnedl C, Holl A, Riedl R, Christopher KB, Pachler C, Urbanic Purkart T, Waltensdorfer A, Munch A, Warnkross H (2014). Effect of high-dose vitamin D_3_ on hospital length of stay in critically ill patients with vitamin D deficiency: the VITdAL-ICU randomized clinical trial. JAMA.

[40] Putzu A, Belletti A, Cassina T, Clivio S, Monti G, Zangrillo A, Landoni G (2016). Vitamin D and outcomes in adult critically ill patients: a systematic review and meta-analysis of randomized trials. J Crit Care.

[41] Weng H, Li JG, Mao Z, Zeng XT (2017). Randomised trials of vitamin D_3_ for critically ill patients in adults: systematic review and meta-analysis with trial sequential analysis. Intensive Care Med.

[42] Langlois PL, Szwec C, D’Aragon F, Heyland DK, Manzanares W. Vitamin D supplementation in the critically ill: a systematic review and meta-analysis. Clin Nutr. doi:10.1016/j.clnu.2017.05.006. 10.1016/j.clnu.2017.05.006 28549527

[43] Amrein K, Martucci G, McNally JD. Letter to the Editor: When not to use meta-analysis: analysing the meta-analyses on vitamin D in critical care. Clin Nutr. doi:10.1016/j.clnu.2017.08.009. 10.1016/j.clnu.2017.08.009 28844336

[44] Moher D, Liberati A, Tetzlaff J, Altman DG (2009). Preferred reporting items for systematic reviews and meta-analyses: the PRISMA statement. PLoS Med.

[45] Nama N, Iliriani K, Xia MY, Chen BP, Zhou LL, Pojsupap S, Kappel C, O’Hearn K, Sampson M, Menon K, McNally JD (2017). A pilot validation study of crowdsourcing systematic reviews: update of a searchable database of pediatric clinical trials of high-dose vitamin D. Transl Pediatr.

[46] Nama N, Menon K, Iliriani K, Pojsupap S, Sampson M, O’Hearn K, Zhou LL, McIntyre L, Fergusson D, McNally JD (2016). A systematic review of pediatric clinical trials of high dose vitamin D. PeerJ.

[47] Sampson M, McGowan J, Cogo E, Grimshaw J, Moher D, Lefebvre C (2009). An evidence-based practice guideline for the peer review of electronic search strategies. J Clin Epidemiol.

[48] Harris PA, Taylor R, Thielke R, Payne J, Gonzalez N, Conde JG (2009). Research electronic data capture (REDCap) – a metadata-driven methodology and workflow process for providing translational research informatics support. J Biomed Inf.

[49] McNally JD, Iliriani K, Pojsupap S, Sampson M, O’Hearn K, McIntyre L, Fergusson D, Menon K (2015). Rapid normalization of vitamin D levels: a meta-analysis. Pediatrics.

[50] Pojsupap S, Iliriani K, Sampaio TZ, O’Hearn K, Kovesi T, Menon K, McNally JD (2015). Efficacy of high-dose vitamin D in pediatric asthma: a systematic review and meta-analysis. J Asthma.

[51] McNally JD, Sampson M, Matheson LA, Hutton B, Little J (2014). Vitamin D receptor (VDR) polymorphisms and severe RSV bronchiolitis: a systematic review and meta-analysis. Pediatr Pulmonol.

[52] United Nations Development Programme. Human Development Index and its components. In: Human development report 2016. http://hdr.undp.org/sites/default/files/2016_human_development_report.pdf. Accessed 23 Oct 2017

[53] Wan X, Wang W, Liu J, Tong T (2014). Estimating the sample mean and standard deviation from the sample size, median, range and/or interquartile range. BMC Med Res Methodol.

[54] Gauthier B, Trachtman H, Di Carmine F, Urivetsky M, Tobash J, Chasalow F, Walco G, Schaeffer J (1990). Hypocalcemia and hypercalcitoninemia in critically ill children. Crit Care Med.

[55] Ayulo M, Katyal C, Agarwal C, Sweberg T, Rastogi D, Markowitz M, Ushay HM (2014). The prevalence of vitamin D deficiency and its relationship with disease severity in an urban pediatric critical care unit. Endocr Regul.

[56] Ebenezer K, Job V, Antonisamy B, Dawodu A, Manivachagan MN, Steinhoff M (2016). Serum vitamin D status and outcome among critically ill children admitted to the pediatric intensive care unit in South India. Indian J Pediatr.

[57] Hebbar KB, Wittkamp M, Alvarez JA, McCracken CE, Tangpricha V (2014). Vitamin D deficiency in pediatric critical illness. J Clin Transl Endocrinol.

[58] Korwutthikulrangsri M, Mahachoklertwattana P, Lertbunrian R, Chailurkit LO, Poomthavorn P (2015). Vitamin D deficiency and adrenal function in critically ill children. J Med Assoc Thai.

[59] Madden K, Feldman HA, Chun RF, Smith EM, Sullivan RM, Agan AA, Keisling SM, Panoskaltsis-Mortari A, Randolph AG (2015). Critically ill children have low vitamin D-binding protein, influencing bioavailability of vitamin D. Ann Am Thorac Soc.

[60] Madden K, Feldman HA, Smith EM, Gordon CM, Keisling SM, Sullivan RM, Hollis BW, Agan AA, Randolph AG (2012). Vitamin D deficiency in critically ill children. Pediatrics.

[61] McNally JD, Doherty DR, Lawson ML, Al-Dirbashi OY, Chakraborty P, Ramsay T, Menon K (2013). The relationship between vitamin D status and adrenal insufficiency in critically ill children. J Clin Endocrinol Metab.

[62] McNally JD, Menon K, Chakraborty P, Fisher L, Williams KA, Al-Dirbashi OY, Doherty DR (2012). The association of vitamin D status with pediatric critical illness. Pediatrics.

[63] Onwuneme C, Carroll A, Doherty D, Bruell H, Segurado R, Kilbane M, Murphy N, McKenna MJ, Molloy EJ (2015). Inadequate vitamin D levels are associated with culture positive sepsis and poor outcomes in paediatric intensive care. Acta Paediatr.

[64] Prasad S, Raj D, Warsi S, Chowdhary S (2015). Vitamin D deficiency and critical illness. Indian J Pediatr.

[65] Rey C, Sanchez-Arango D, Lopez-Herce J, Martinez-Camblor P, Garcia-Hernandez I, Prieto B, Pallavicini Z (2014). Vitamin D deficiency at pediatric intensive care admission. J Pediatr (Rio J).

[66] Dayal D, Kumar S, Sachdeva N, Kumar R, Singh M, Singhi S (2014). Fall in Vitamin D levels during hospitalization in children. Int J Pediatr.

[67] Ponnarmeni S, Kumar Angurana S, Singhi S, Bansal A, Dayal D, Kaur R, Patial A, Verma AS (2016). Vitamin D deficiency in critically ill children with sepsis. Paediatr Int Child Health.

[68] McNally JD, Menon K, Lawson ML, Williams K, Doherty DR (2015). 1,25-Dihydroxyvitamin D levels in pediatric intensive care units: risk factors and association with clinical course. J Clin Endocrinol Metab.

[69] Rippel C, South M, Butt WW, Shekerdemian LS (2012). Vitamin D status in critically ill children. Intensive Care Med.

[70] García-Soler P, Morales-Martínez A, Rosa-Camacho V, Lillo-Muñoz JA, Milano-Manso G (2017). Vitamin D deficiency and morbimortality in critically ill paediatric patients [in Spanish]. An Pediatr (Barc).

[71] Bustos BR, Rodriguez-Nunez I, Pena Zavala R, Soto GG (2016). Vitamin D deficiency in children admitted to the paediatric intensive care unit [in Spanish]. Rev Chil Pediatr.

[72] Shah SK, Kabra SK, Gupta N, Pai G, Lodha R (2016). Vitamin D deficiency and parathyroid response in critically-ill children: association with illness severity and clinical outcomes. Indian Pediatr.

[73] Sankar J, Lotha W, Ismail J, Anubhuti C, Meena RS, Sankar MJ (2016). Vitamin D deficiency and length of pediatric intensive care unit stay: a prospective observational study. Ann Intensive Care.

[74] Langlois K, Greene-Finestone L, Little J, Hidiroglou N, Whiting S (2010). Vitamin D status of Canadians as measured in the 2007 to 2009 Canadian Health Measures Survey. Health Rep.

[75] El Hayek J, Pham TT, Finch S, Hazell TJ, Jean-Philippe S, Vanstone CA, Agellon S, Rodd C, Rauch F, Weiler HA (2013). Vitamin D status in Montreal preschoolers is satisfactory despite low vitamin D intake. J Nutr.

[76] Han YY, Forno E, Celedón JC (2017). Vitamin D insufficiency and asthma in a US nationwide study. J Allergy Clin Immunol Pract.

[77] Soininen S, Eloranta AM, Lindi V, Venalainen T, Zaproudina N, Mahonen A, Lakka TA (2016). Determinants of serum 25-hydroxyvitamin D concentration in Finnish children: the Physical Activity and Nutrition in Children (PANIC) study. Br J Nutr.

[78] Perron RM, Lee P (2013). Efficacy of high-dose vitamin D supplementation in the critically ill patients. Inflamm Allergy Drug Targets.

[79] Dhaliwal R, Cahill N, Lemieux M, Heyland DK (2014). The Canadian critical care nutrition guidelines in 2013: an update on current recommendations and implementation strategies. Nutr Clin Pract.

[80] Krueger M, Puthothu B, Heinze J, Forster J, Heinzmann A (2006). Genetic polymorphisms of adhesion molecules in children with severe RSV-associated diseases. Int J Immunogenet.

[81] Balluffi A, Kassam-Adams N, Kazak A, Tucker M, Dominguez T, Helfaer M (2004). Traumatic stress in parents of children admitted to the pediatric intensive care unit. Pediatr Crit Care Med.

[82] Colville G, Darkins J, Hesketh J, Bennett V, Alcock J, Noyes J (2009). The impact on parents of a child’s admission to intensive care: integration of qualitative findings from a cross-sectional study. Intensive Crit Care Nurs.

[83] Amrein K, Christopher KB, McNally JD (2015). Understanding vitamin D deficiency in intensive care patients. Intensive Care Med.

[84] Christopher KB (2015). Vitamin D, supplementation in the ICU patient. Curr Opin Clin Nutr Metab Care.

[85] McNally JD, Amrein K (2016). Vitamin D deficiency in pediatric critical care. J Pediatr Intensive Care.

[86] Lucas RM, Ponsonby AL, Dear K, Valery PC, Taylor B, van der Mei I, McMichael AJ, Pender MP, Chapman C, Coulthard A (2013). Vitamin D status: multifactorial contribution of environment, genes and other factors in healthy Australian adults across a latitude gradient. J Steroid Biochem Mol Biol.

[87] Lee P (2011). Vitamin D, metabolism and deficiency in critical illness. Best Pract Res Clin Endocrinol Metab.

[88] Hiemstra TF, Casian A, Boraks P, Jayne DR, Schoenmakers I (2014). Plasma exchange induces vitamin D deficiency. QJM.

[89] McNally JD, Menon K, Chakraborty P, Fisher L, Williams KA, Al-Dirbashi OY, Girolamo T, Maharajh G, Doherty DR (2013). Impact of anesthesia and surgery for congenital heart disease on the vitamin d status of infants and children: a prospective longitudinal study. Anesthesiology.

[90] Reid D, Toole BJ, Knox S, Talwar D, Harten J, O’Reilly DS, Blackwell S, Kinsella J, McMillan DC, Wallace AM (2011). The relation between acute changes in the systemic inflammatory response and plasma 25-hydroxyvitamin D concentrations after elective knee arthroplasty. Am J Clin Nutr.

[91] Jayashree M, Ismail J (2016). Vitamin D deficiency in critically ill children: bystander or culprit?. Indian J Pediatr.

