# Supplementary material for: Vitamin D deficiency in critically ill children: a systematic review and meta-analysis
Source: Crit Care. 2017 Nov 23;21:287. doi: 10.1186/s13054-017-1875-y (PMC5701429; doi:10.1186/s13054-017-1875-y)
Supplement: Supplementary file 2 — Electronic search strategy used for this systematic review. (PDF 452 kb) [file 13054_2017_1875_MOESM2_ESM.pdf]

Supplemental Digital Content 2: Electronic Search Strategy

MEDLINE

1 exp Vitamin d/

2 (vitamin adj (d or d2 or d3)).tw.

3 Calcifediol/

4 calcidiol.tw.

5 Ergocalciferols/

6 Ergocalciferol$.tw.

7 Cholecalciferol/

8 Cholecalciferol$.tw.

9 calciferol.tw.

10 (25-hydroxyvitamin D or 25-hydroxy vitamin d or Plasma vitamin D).tw.

11 64719-49-9.rn.

12 25OHD3.tw.

13 "25(OH)D3".tw.

14 25-OHD3.tw.

15 "25-(OH)D3".tw.

16 25OHD.tw.

17 "25(OH)D".tw.

18 25-OHD.tw.

19 "25-(OH)D".tw.

20 (25-hydroxycholecalciferol or 25-hydroxyergocalciferol).tw.

21 plasma calcidiol.tw.

22 Vitamin D Deficiency/dh, dt

23 or/1-22

24 Intensive Care/

25 Intensive Care Units/ or exp Intensive Care Units, Pediatric/

26 exp Critical Care/

27 Critical Illness/

28 (PICU or PCCU or NICU).tw.

29 critical* ill*.tw.

30 intensive care.tw.

31 exp Mortality/

32 mo.fs.

33 exp Death/

34 Shock, septic/

35 (mortality or septic shock or death*).tw.

36 (Paediatric Index of Mortality or PRISM III).tw.

37 (sequential organ failure assessment or SOFA).tw.

38 (multiple organ dysfunction syndrome or MODS).tw.

39 (Pediatric Logistic Organ Dysfunction or PELOD).tw.

40 Inotropes.tw.

41 exp Vasoconstrictor Agents/

42 exp Respiration, Artificial/

43 Mechanical* vent*.tw.

44 Extracorporeal Membrane Oxygenation/

45 (extracorporeal membrane oxygenation or ECMO).tw.

46 or/24-45

47 (child* or adolescent* or infan*).mp.

48 23 and 46 and 47

49 remove duplicates from 48

PUBMED

#6	Search **(((((((((((((((((((((((((((vitamin D[MeSH Terms]) OR (("vitamin d"[Text Word] OR "vitamin d2"[Text Word] OR "vitamin d3"[Text Word]))) OR Calcifediol[MeSH Terms]) OR calcidiol*[Text Word]) OR Ergocalciferols[MeSH Terms]) OR Ergocalciferol*[Text Word]) OR Cholecalciferol[mh:noexp]) OR Cholecalciferol*[Text Word]) OR calciferol[Text Word]) OR (("25-hydroxyvitamin D"[Text Word] OR "25-hydroxy vitamin d"[Text Word]))) OR "plasma vitamin D"[Text Word]) OR 64719-49-9[EC/RN Number]) OR 25OHD3[Text Word]) OR "25(OH)D3"[Text Word]) OR "25-OHD3"[Text Word]) OR "25-(OH)D3"[Text Word]) OR 25OHD[Text Word]) OR "25(OH)D"[Text Word]) OR "25-OHD"[Text Word]) OR "25-(OH)D"[Text Word]) OR "25-hydroxycholecalciferol"[Text Word]) OR "25-hydroxyergocalciferol"[Text Word]) OR plasma calcidiol[Text Word]) OR Vitamin D Deficiency/diet therapy[MeSH Terms]) OR Vitamin D Deficiency/drug therapy[MeSH Terms])) AND (((((((((((((((((((((((((Intensive Care[mh:noexp]) OR Intensive Care Units[mh:noexp]) OR Intensive Care Units, Pediatric[MeSH Terms]) OR Critical Illness[mh:noexp]) OR Critical Care[MeSH Terms]) OR ((PICU[Text Word] OR PCCU[Text Word] OR NICU[Text Word]))) OR critical* AND ill*[Text Word]) OR intensive care[Text Word]) OR mortality[MeSH Terms]) OR mortality[MeSH Subheading]) OR death[MeSH Terms]) OR Shock, Septic[MeSH Terms]) OR ((mortality[Text Word] OR septic shock[Text Word] OR death*[Text Word]))) OR ((Paediatric Index of Mortality[Text Word] OR PRISM III[Text Word]))) OR ((sequential organ failure assessment[Text Word] OR SOFA[Text Word]))) OR ((Multiple organ dysfunction syndrome OR MODS[MeSH Terms]))) OR ((Pediatric Logistic Organ Dysfunction[Text Word] OR PELOD[Text Word]))) OR Inotropes[Text Word]) OR Vasoconstrictor Agents[MeSH Terms]) OR Respiration, Artificial[MeSH Terms]) OR "Mechanical* vent*"[Text Word]) OR Extracorporeal Membrane Oxygenation[MeSH Terms]) OR ((extracorporeal membrane oxygenation[Text Word] OR ECMO[Text Word])))) OR ((crit* AND ill*[Text Word]) OR mech* AND vent*[Text Word]))) AND (Infan* OR newborn* OR new-born* OR neonat* OR baby OR baby* OR babies OR toddler* OR minors OR minors* OR boy OR boys OR boyfriend OR boyhood OR girl* OR kid OR kids OR child OR child* OR children* OR schoolchild* OR schoolchild OR school child[tiab] OR school child*[tiab] OR adolescen* OR juvenil* OR youth* OR teen* OR under*age* OR pubescen* OR pediatrics[mh] OR pediatric* OR paediatric* OR peadiatric* OR school[tiab] OR school*[tiab])**
#5	Search **Infan* OR newborn* OR new-born* OR neonat* OR baby OR baby* OR babies OR toddler* OR minors OR minors* OR boy OR boys OR boyfriend OR boyhood OR girl* OR kid OR kids OR child OR child* OR children* OR schoolchild* OR schoolchild OR school child[tiab] OR school child*[tiab] OR adolescen* OR juvenil* OR youth* OR teen* OR under*age* OR pubescen* OR pediatrics[mh] OR pediatric* OR paediatric* OR peadiatric* OR school[tiab] OR school*[tiab]**
#4	Search **((((((((((((((((((((((((Intensive Care[mh:noexp]) OR Intensive Care Units[mh:noexp]) OR Intensive Care Units, Pediatric[MeSH Terms]) OR Critical Illness[mh:noexp]) OR Critical Care[MeSH Terms]) OR**

	**((PICU[Text Word] OR PCCU[Text Word] OR NICU[Text Word]))) OR critical* AND ill*[Text Word]) OR intensive care[Text Word]) OR mortality[MeSH Terms]) OR mortality[MeSH Subheading]) OR death[MeSH Terms]) OR Shock, Septic[MeSH Terms]) OR ((mortality[Text Word] OR septic shock[Text Word] OR death*[Text Word]))) OR ((Paediatric Index of Mortality[Text Word] OR PRISM III[Text Word]))) OR ((sequential organ failure assessment[Text Word] OR SOFA[Text Word]))) OR ((Multiple organ dysfunction syndrome OR MODS[MeSH Terms]))) OR ((Pediatric Logistic Organ Dysfunction[Text Word] OR PELOD[Text Word]))) OR Inotropes[Text Word]) OR Vasoconstrictor Agents[MeSH Terms]) OR Respiration, Artificial[MeSH Terms]) OR "Mechanical* vent*"[Text Word]) OR Extracorporeal Membrane Oxygenation[MeSH Terms]) OR ((extracorporeal membrane oxygenation[Text Word] OR ECMO[Text Word])))) OR ((crit* AND ill*[Text Word]) OR mech* AND vent*[Text Word])**
#3	Search **(crit* AND ill*[Text Word]) OR mech* AND vent*[Text Word]**
#2	Search **((((((((((((((((((((Intensive Care[mh:noexp]) OR Intensive Care Units[mh:noexp]) OR Intensive Care Units, Pediatric[MeSH Terms]) OR Critical Illness[mh:noexp]) OR Critical Care[MeSH Terms]) OR ((PICU[Text Word] OR PCCU[Text Word] OR NICU[Text Word]))) OR critical* AND ill*[Text Word]) OR intensive care[Text Word]) OR mortality[MeSH Terms]) OR mortality[MeSH Subheading]) OR death[MeSH Terms]) OR Shock, Septic[MeSH Terms]) OR ((mortality[Text Word] OR septic shock[Text Word] OR death*[Text Word]))) OR ((Paediatric Index of Mortality[Text Word] OR PRISM III[Text Word]))) OR ((sequential organ failure assessment[Text Word] OR SOFA[Text Word]))) OR ((Multiple organ dysfunction syndrome OR MODS[MeSH Terms]))) OR ((Pediatric Logistic Organ Dysfunction[Text Word] OR PELOD[Text Word]))) OR Inotropes[Text Word]) OR Vasoconstrictor Agents[MeSH Terms]) OR Respiration, Artificial[MeSH Terms]) OR "Mechanical* vent*"[Text Word]) OR Extracorporeal Membrane Oxygenation[MeSH Terms]) OR ((extracorporeal membrane oxygenation[Text Word] OR ECMO[Text Word]))**
#1	Search **((((((((((((((((((((((vitamin D[MeSH Terms]) OR (("vitamin d"[Text Word] OR "vitamin d2"[Text Word] OR "vitamin d3"[Text Word]))) OR Calcifediol[MeSH Terms]) OR calcidiol*[Text Word]) OR Ergocalciferols[MeSH Terms]) OR Ergocalciferol*[Text Word]) OR Cholecalciferol[mh:noexp]) OR Cholecalciferol*[Text Word]) OR calciferol[Text Word]) OR (("25-hydroxyvitamin D"[Text Word] OR "25-hydroxy vitamin d"[Text Word]))) OR "plasma vitamin D"[Text Word]) OR 64719-49-9[EC/RN Number]) OR 25OHD3[Text Word]) OR "25(OH)D3"[Text Word]) OR "25-OHD3"[Text Word]) OR "25-(OH)D3"[Text Word]) OR 25OHD[Text Word]) OR "25(OH)D"[Text Word]) OR "25-OHD"[Text Word]) OR "25-(OH)D"[Text Word]) OR "25-hydroxycholecalciferol"[Text Word]) OR "25-hydroxyergocalciferol"[Text Word]) OR plasma calcidiol[Text Word]) OR Vitamin D Deficiency/diet therapy[MeSH Terms]) OR Vitamin D Deficiency/drug therapy[MeSH Terms]**

EMBASE

1 exp vitamin D/

2 (vitamin adj (d or d2 or d3)).tw.

3 calcifediol/

4 calcidiol.tw.

5 ergocalciferol/

6 Ergocalciferol$.tw.

7 colecalciferol/

8 cholecalciferol$.tw.

9 (25-hydroxyvitamin D or 25-hydroxy vitamin d or Plasma vitamin D).tw.

10 64719-49-9.rn.

11 25OHD3.tw.

12 "25(OH)D3".tw.

13 25-OHD3.tw.

14 "25-(OH)D3".tw.

15 25OHD.tw.

16 "25(OH)D".tw.

17 25-OHD.tw.

18 "25-(OH)D".tw.

19 (25-hydroxycholecalciferol or 25-hydroxyergocalciferol).tw.

20 plasma calcidiol.tw.

21 vitamin D deficiency/dt [Drug Therapy]

22 vitamin D deficiency/ and diet therapy/

23 or/1-22

24 intensive care/

25 intensive care unit/

26 newborn intensive care/

27 critical illness/

28 (PICU or PCCU or NICU).tw.

29 (critical* adj (care or ill*)).tw.

30 intensive care.tw.

31 mortality/

32 childhood mortality/ or infant mortality/

33 newborn mortality/

34 child death/ or newborn death/

35 death/

36 septic shock/

37 (mortality or septic shock or death*).tw.

38 (Paediatric Index of Mortality or PRISM III).tw.

39 sequential organ failure assessment score/

40 (sequential organ failure assessment or SOFA).tw.

41 multiple organ failure/

42 (multiple organ dysfunction syndrome or MODS).tw.

43 (Pediatric Logistic Organ Dysfunction or PELOD).tw.

44 Inotropes.tw.

45 exp vasoconstrictor agent/

46 exp artificial ventilation/

47 mechanical* ven*.tw.

48 extracorporeal oxygenation/

49 (extracorporeal membrane oxygenation or ECMO).tw.

50 or/24-49

51 23 and 50

52 (Infan* or newborn* or new-born* or neonat* or baby or baby* or babies or toddler* or minors or minors* or boy or boys or girl* or kids or child or child* or children* or schoolchild* or schoolchild).mp.or school child.ti,ab. or school child*.ti,ab. or (adolescen* or juvenil* or youth* or teen* or under*age* or pubescen*).mp. orexp pediatrics/ or (pediatric* or paediatric* or peadiatric*).mp. orschool.ti,ab. or school*.ti,ab.

53 51 and 52

54 remove duplicates from 53

55 limit 54 to embase

COCHRANE CENTRAL REGISTER OF CONTROLLED TRIALS

1 (vitamin adj (d or d2 or d3)).tw.

2 (calcifediol or calcidiol or ergocalciferol$ or cholecalciferol$ or calciferol$).tw.

3 (vitamin adj d adjdeficienc$).tw.

4 or/1-3

5 (critical adj (care or ill$)).tw.

6 (critical adj2 (care or ill$)).tw.

7 (intensive adj2 care$).tw.

8 (PICU or PCCU).mp.or NICU.tw.

9 mortality.tw.

10 death$.tw.

11 septic shock.tw.

12 "Paediatric Index of Mortality".tw.

13 PRISM III.tw.

14 (sequential organ failure assessment or SOFA).tw.

15 (multiple organ dysfunction syndrome or MODS).tw.

16 (Pediatric Logistic Organ Dysfunction or PELOD).tw.

17 Inotropes.tw.

18 vasoconstrictor$.tw.

19 (artificial adj2 respir$).tw.

20 (mechanical$ adj2 vent$).tw.

21 extracorporeal membrane oxygen$.tw.

22 (extracorporeal adj2 membrane adj2 oxygen$).tw.

23 ECMO.tw.

24 or/5-23

25 (Infan* or newborn* or new-born* or perinat* or neonat* or baby or baby* or babies or toddler* or minors or minors* or boy or boys or boyfriend or boyhood or girl* or kid or kids or child or child* or children* or schoolchild* or schoolchild).mp.or school child.ti,ab. or school child*.ti,ab. or (adolescen* or juvenil* or youth* or teen* or under*age* or pubescen*).mp. orexp pediatrics/ or (pediatric* or paediatric* or peadiatric*).mp. orschool.ti,ab. or school*.ti,ab.

26 4 and 24 and 25
