# Supplementary material for: Vitamin D deficiency in critically ill children: a systematic review and meta-analysis
Source: Crit Care. 2017 Nov 23;21:287. doi: 10.1186/s13054-017-1875-y (PMC5701429; doi:10.1186/s13054-017-1875-y)
Supplement: Supplementary file 3 — Table summarizing the screening criteria used for level 1 and level 2 screening. (PDF 165 kb) [file 13054_2017_1875_MOESM3_ESM.pdf]

Supplemental Digital Content 3: Level 1 and Level 2 screening criteria

	**Level 1 (**Article may be excluded if it is)
1	• A review article, letter, editorial<br>• A systematic review<br>• Surveys<br>• Interventional study<br>• Study population is animal or cell line<br>• Study population has a specific genetic based disease<br>• Study describes less than 10 patients<br>• There is no mention of vitamin D levels, vitamin D status or vitamin D deficiency in the abstract (exclude those that only mention vitamin D binding protein or vitamin D receptors)<br>• No mention of critical illness, intensive care, mortality/death, mechanical ventilation, cardiovascular support (vasopressors, inotrope sore, ECMO, etc) in the abstract
	**Level 2 (article should be excluded if)**
2	• Does not include children (=<18 years)<br>• Includes children over 21 years but does not report data separately for children
3	• No patients admitted to PICU
4	Does not report on vitamin D status using 25OHD level<br>Vitamin D levels were measured prior to death (e.g. does not represent a SIDS or related syndrome where patients were dead)
5	• Does not report on one or more of the following ICU outcomes<br>○ Mortality (PICU or hospital)<br>○ Mechanical ventilation (e.g. intubation, duration of MV)<br>○ Vasoactive agent use (e.g. inotrope score, catecholamines, vasopressors)<br>○ PICU Illness severity score (e.g. PRISM, PELOD, PIM)
6	• Study type other than observational cohort or case control study
7	• Meets above, but enrolled a specific disease process or surgical procedure (ALRI, Cardiac surgery, prematurity

PICU – pediatric intensive care unit; ECMO – Extracorporeal membrane oxygenation; SIDS – sudden infant death syndrome; ICU – intensive care unit; MV – mechanical ventilation; PRISM – Pediatric Risk of Mortality; PELOD - Pediatric Logistic Organ Dysfunction; PIM –Pediatric Risk of Mortality; ALRI – Acute lower respiratory infection
