# Supplementary material for: Vitamin D deficiency in critically ill children: a systematic review and meta-analysis
Source: Crit Care. 2017 Nov 23;21:287. doi: 10.1186/s13054-017-1875-y (PMC5701429; doi:10.1186/s13054-017-1875-y)
Supplement: Supplementary file 4 — Newcastle-Ottawa Scale adapted for this systematic review. (PDF 352 kb) [file 13054_2017_1875_MOESM4_ESM.pdf]

Supplemental Digital Content 4: Newcastle Ottawa Scale (adapted for this systematic review)

Selection

1) Representativeness of the exposed cohort
    - a) truly representative of the average ICU patient (*point given)
    - b) somewhat representative of the average ICU patient (*point given)
    - c) selected group
    - d) no description of the derivation of the cohort

2) Selection of the non- exposed cohort
    - a) drawn from the same community as the exposed cohort (*point given)
    - b) drawn from a different source
    - c) no description of the derivation of the non-exposed cohort

3) Ascertainment of exposure (i.e. of being vitamin D deficient)
    - a) secure record - reliable assay for vitamin D status (*point given)
    - b) Vitamin D status determined well before or after ICU admission
    - c) no report of accuracy of vitamin D assay
    - d) no description

4) Demonstration that outcome(s) of interest (e.g. mortality , LOS) was not present at start of study
    - a) yes (*point given)
    - b) no (for example if determined vitamin D level at time of discharge)

Comparability

1) Comparability of cohorts on the basis of the design or analysis
    - a) study controls for Age (*point given)
    - b) study controls for any additional factor ***** (point given)
    - will be study dependent, but could consider things like e.g. illness severity at admission, other measure of nutritional status)

Outcome

1) Assessment of outcome
    - a) independent assessment (*point given)
    - b) record linkage (*point given)
    - c) self-report
    - d) no description

2) Was follow-up long enough for outcomes to occur
    - a) yes – in hospital mortality (*point given)
    - b) no (indicate no if mortality was only PICU mortality or unclear)

3) Adequacy of follow up of cohorts
    - a) complete follow up - all subjects accounted for(*point given)
    - b) subjects lost to follow up unlikely to introduce bias < 20 % (*point given)
    - c) follow up rate < 80 % and no description of those lost
    - d) no statement
