# Supplementary material for: Vitamin D deficiency in critically ill children: a systematic review and meta-analysis
Source: Crit Care. 2017 Nov 23;21:287. doi: 10.1186/s13054-017-1875-y (PMC5701429; doi:10.1186/s13054-017-1875-y)
Supplement: Supplementary file 5 — Comparison of vitamin D status at PICU admission (or first day in PICU) of critically ill children and healthy control subjects. Table summarizing vitamin D status at PICU admission (or first day in PICU) of critically ill children and healthy control subjects, and the pooled mean difference in 25(OH)D levels between the PICU and control cohorts. (PDF 78 kb) [file 13054_2017_1875_MOESM5_ESM.pdf]

Supplemental Digital Content 5: Comparison of Vitamin D status at PICU admission (or first day in PICU) for critically ill children and healthy controls

Trial, Year	Threshold VDD (nmol/L)	PICU cohort		Control cohort					
		%VDD	Average 25(OH)D (nmol/L) [b]	(n[a])	%VDD	Average 25(OH)D (nmol/L) [b]	Difference in 25(OH)D (nmol/L)	95 %CI (UL, LL)	P value
Gauthier, 1990	22.5	NR	66.8[c] (±20)	12	NR	82.5[c] (±25)	-15.7	-33.8, 2.4	0.09
Hebbar, 2014	50	61	59[c] (±43)	42	23.9	99[c] (±55)	-40	-58.57, -21.4	0.000
Rey, 2014	50	30	65 (48 – 89.5)	289	16%	76.3 (58-96.5)	-19.5	-25.2, -13.8	0.000
Korwutthikulrangsri, 2015	50	78	41.5 (33.3-48.8)	36	19.5	61[c] (±12)	-19.0	-30.4, -7.62	0.000
Onwuneme, 2015	50	59	47[c] (±29)	30	NR	66[c] (±26)	-19.8	-27.5, -12.0	0.001
Ponnarmeni, 2016	50	51	49.25 (±30)	338	40.2%	68.7[c] (±40)	-11.3	-17.0, -5.6	0.000
**Pooled Mean Difference**							**-17.3**	**-14.0, -20.6**	**0.000**

[a]Total number of enrolled patients, by group, for which a measure of vitamin D status was available. [b]Reported as median, unless otherwise specified as mean[c]. Distribution reported as either IQR (XX-XX) or Standard Deviation (±XX). 25(OH)D = 25 hydroxyvitamin D; CI = Confidence Interval; NR = Not reported; PICU = Pediatric Intensive Care Unit; VDD = Vitamin D deficiency. [c]Data provided by authors outside of publication.
