# Supplementary material for: Vitamin D deficiency in critically ill children: a systematic review and meta-analysis
Source: Crit Care. 2017 Nov 23;21:287. doi: 10.1186/s13054-017-1875-y (PMC5701429; doi:10.1186/s13054-017-1875-y)
Supplement: Supplementary file 6 — Table summarizing the demographics and risk factors for vitamin D deficiency evaluated by each study included in this systematic review. (PDF 77 kb) [file 13054_2017_1875_MOESM6_ESM.pdf]

Supplemental Digital Content 6: Demographics and risk factors evaluated in each study

Study name (First author, year)	Weight Nutrition	Bone	Renal	Liver	Malabsorption	Age	Admission type	Vitamin D Supplement	Race, skin color	Season	Preexisting disease
Gauthier, 1990			EC			No					
Madden, 2012	No					Yes	No	Yes	Yes	Yes	No
McNally, 2012	Yes			No		No	Yes			No	No
Rippel, 2012		EC	EC	EC		No	Yes		No	No	
Ayulo, 2014	Yes					Yes	No		Yes	No	
Dayal, 2014	Yes	No	EC	EC	EC	No	No	No	No		No
Hebbar, 2014			EC		EC	No			Yes	Yes	No
Rey, 2014	Yes					Yes	Yes				No
Korwutthikulrangsri, 2015				EC		No	No	No			No
Onwuneme, 2015	No					No		Yes		No	No
Prasad, 2015	No					No				No	
Ebenezer, 2016	No					Yes	No		No	No	No
Ponnarmeni, 2016	Yes					No				No	EC
Bustos, 2016	No		EC	EC		No	No			Yes	No
Garcia-Soler, 2016	Yes		EC		EC	Yes	Yes	EC	Yes	Yes	No
Sankar, 2016	No	EC	EC			No	No	EC			
Shah, 2016	No	EC	EC			Yes	No			Yes	No

A blank entry means that the study did not evaluate for a relationship between vitamin D status and that patient characteristics. Yes = Statistically significant relationship observed in at least one analyses. No = Statistical relationship not observed. Abbreviations: EC = Was part of exclusion criteria
