# Supplementary figures and images for: Vitamin D deficiency in critically ill children: a systematic review and meta-analysis
Source: Crit Care. 2017 Nov 23;21:287. doi: 10.1186/s13054-017-1875-y (PMC5701429; doi:10.1186/s13054-017-1875-y)

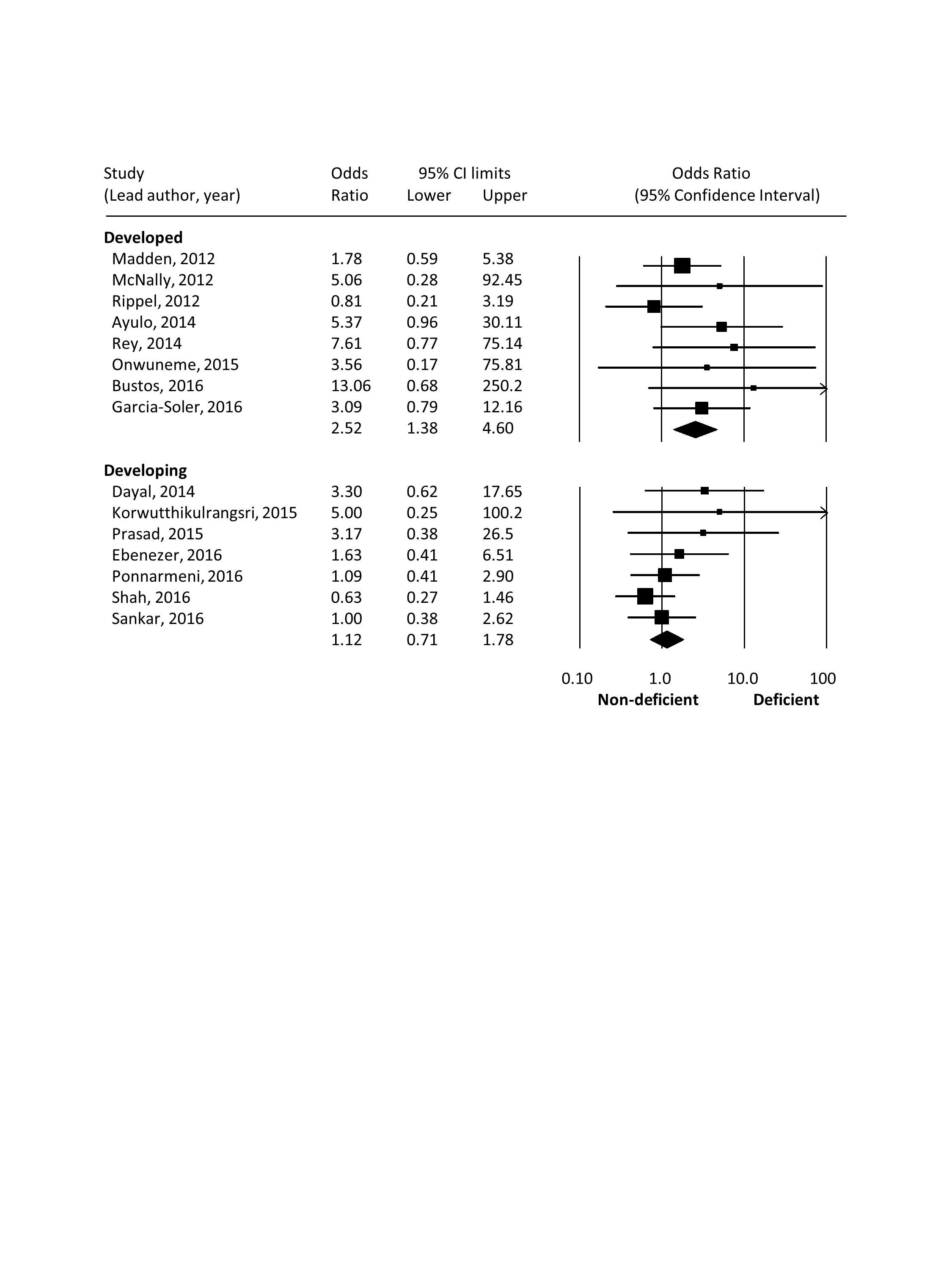

Supplement: Supplementary file 7 — Association of vitamin D deficiency and mortality by country type. Figure showing association of vitamin D deficiency and mortality in developed and developing countries. (TIF 218 kb) [file 13054_2017_1875_MOESM7_ESM.tif]

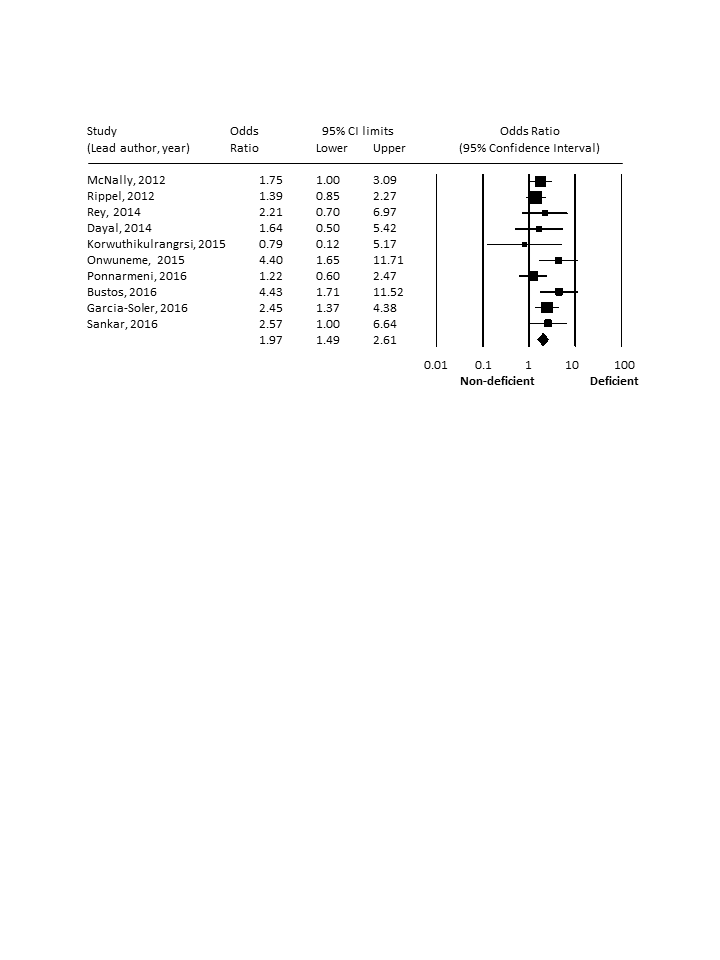

Supplement: Supplementary file 8 — Vitamin D deficiency and clinical outcomes in the PICU. Figure showing association of vitamin D deficiency with vasopressor use. (TIF 74 kb) [file 13054_2017_1875_MOESM8_ESM.tif]

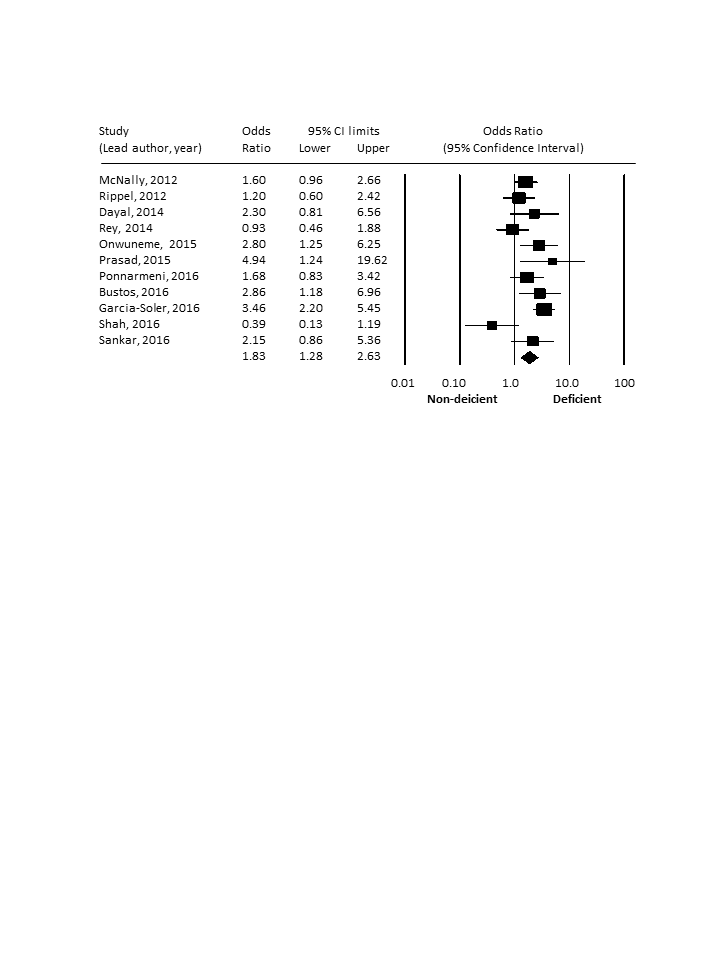

Supplement: Supplementary file 9 — Vitamin D deficiency and clinical outcomes in the PICU. Figure showing association of vitamin D deficiency with mechanical ventilation. (TIF 74 kb) [file 13054_2017_1875_MOESM9_ESM.tif]

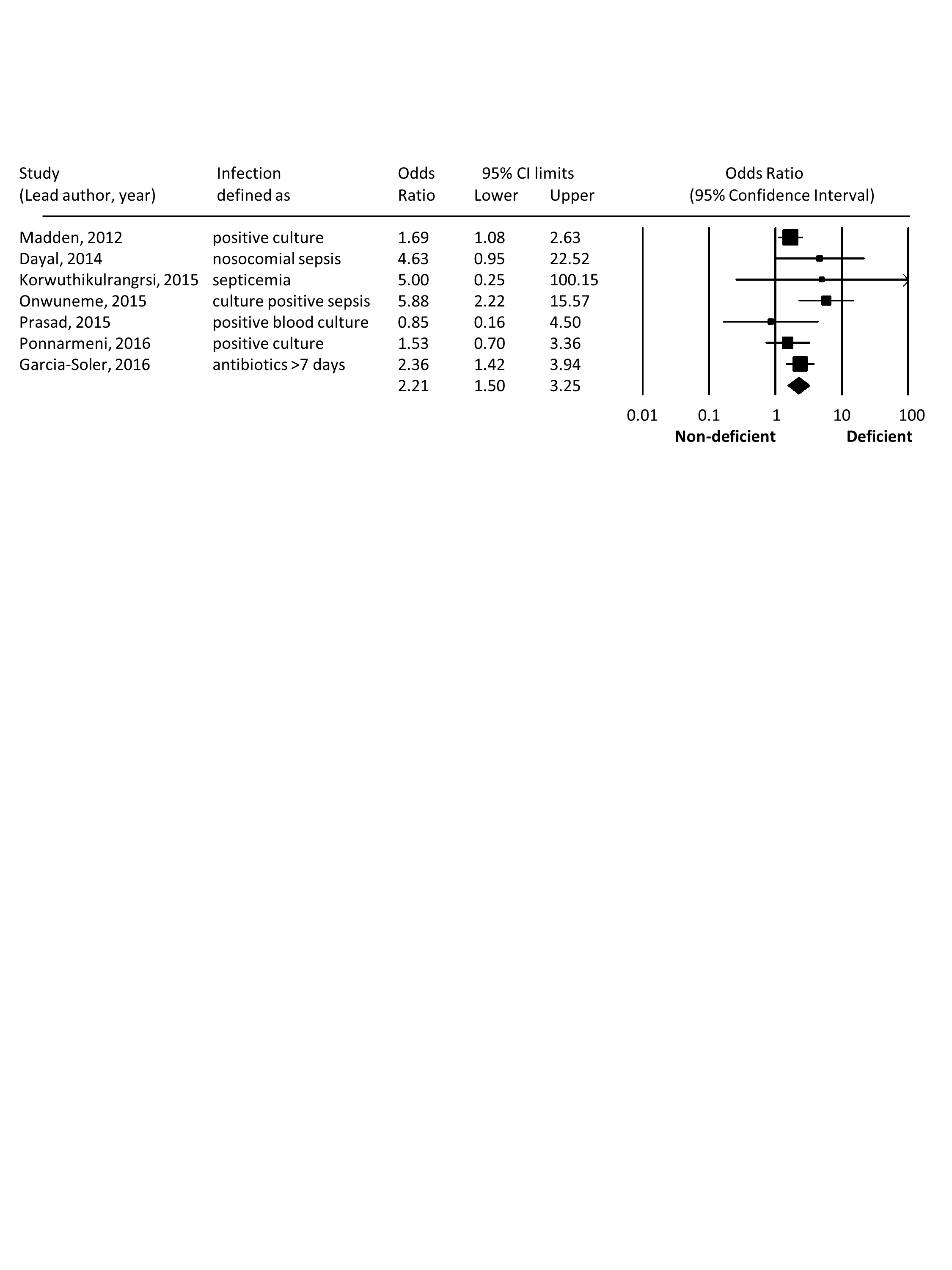

Supplement: Supplementary file 10 — Vitamin D deficiency and clinical outcomes in the PICU. Figure showing association of vitamin D deficiency with confirmed bacterial or nosocomial infection. (TIF 175 kb) [file 13054_2017_1875_MOESM10_ESM.tif]
